# LncRNA SATB2-AS1 overexpression represses the development of hepatocellular carcinoma through regulating the miR-3678-3p/GRIM-19 axis

**DOI:** 10.1186/s12935-023-02901-1

**Published:** 2023-04-28

**Authors:** Jiang Huang, Yunfang Yang, Fulan Zhao, Zhuo Zhang, Jian Deng, Wei Lu, Xian Jiang

**Affiliations:** 1 Department of Pharmacy, The Affiliated Traditional Chinese Medicine Hospital of Southwest Medical University, Luzhou, 646000 Sichuan China; 2 Department of Neurology, The Affiliated Traditional Chinese Medicine Hospital of Southwest Medical University, Luzhou, 646000 Sichuan China; 3 Department of Pharmacology, Southwest Medical University, Luzhou, 646000 Sichuan China; 4 Department of Emergency, Luzhou People’s Hospital, Luzhou, 646000 Sichuan China; 5 Department of Anesthesiology, Luzhou People’s Hospital, No. 316, Jiugu Avenue 2, Jiangyang District, Luzhou, 646000 Sichuan China

**Keywords:** Hepatocellular carcinoma, SATB2-AS1, miR-3678-3p, GRIM-19, Proliferation, Metastasis

## Abstract

Hepatocellular carcinoma (HCC) is a malignancy worldwide with one of the worst prognoses. Emerging studies have revealed that long noncoding RNAs (lncRNAs) contribute to HCC progression. This research probes the expression and regulatory effect of lncRNA SATB2-AS1 on HCC development. Reverse transcription-polymerase chain reaction (RT-PCR) was applied to measure the SATB2-AS1 profile in HCC tissues and adjacent non-tumor tissues. The impact of SATB2-AS1, miR-3678-3p, or GRIM-19 on HCC cell proliferation, growth, migration, invasion, and apoptosis was determined by gain- and loss-of-function experiments. The results revealed that SATB2-AS1 was downregulated in HCC tissues, and its lower levels were related to higher tumor staging and poorer prognosis of HCC patients. SATB2-AS1 overexpression repressed HCC cell proliferation, induced G1 arrest, and apoptosis, and inhibited migration, invasion, and epithelial-mesenchymal transition (EMT). Mechanistically, SATB2-AS1 inactivated STAT3/HIF-1α and strengthened GRIM-19 expression. After knocking down GRIM-19 with small interfering RNA (siRNA), the malignant phenotypes of HCC cells were enhanced. Further bioinformatics analysis showed that miR-3678-3p was targeted by SATB2-AS1. The dual-luciferase reporter assay, RNA immunoprecipitation (RIP) experiment, and Fluorescence in situ Hybridization (FISH) test confirmed that SATB2-AS1 sponged miR-3678-3p and the latter targeted GRIM-19. The rescue experiments showed that miR-3678-3p aggravated the malignant behaviors of HCC cells, whereas SATB2-AS1 overexpression reversed miR-3678-3p-mediated effects. Inhibition STAT3 promoted SATB2-AS1 and GRIM-19 expression, and reduced miR-3678-3p level. Activation STAT3 exerted opposite effects. Overall, this study confirmed that SATB2-AS1 is a potential prognostic biomarker for HCC and regulates HCC devolvement by regulating the miR-3678-3p/GRIM-19/STAT3/HIF-1α pathway.

## Introduction

Hepatocellular carcinoma (HCC) is one of the leading causes of cancer-associated mortality worldwide, especially in Asia and Africa [1–3]. It is most common in elderly patients and age is directly correlated with the incidence rates of HCC until approximately 75 years of age [3]. Currently, ultrasound (US) and serum alpha-fetoprotein (AFP) tests are the most frequently used screening methods for high-risk groups [4]. However, due to the poor efficacy of the AFP test and the high dependence of the US on operators, the efficiency of early screening and diagnosis is not ideal. Accordingly, advanced HCC patients have a poor prognosis [5]. Hence, it’s vital to explore the pathogenesis of HCC and search for new diagnostic markers and identify useful therapeutic targets.

Long noncoding RNAs (lncRNAs) are longer than 200 nucleotides and are not translated into proteins. Reports have stated that lncRNAs exert a tumor-suppressive or carcinogenic role in diversified malignant cancers [6]. Taking lncRNA GAS5-AS1 as an example, it downregulates TUSC2 expression by sponging miR-106b-5p, thus suppressing glioma cell proliferation and migration and promoting cell apoptosis [7]. LncRNA PVT1 activates the signal transductor and transcriptional activator 3 (STAT3), thereby inducing cell cycle processes, and promoting the proliferation and metastasis of HCC cells [8]. SATB2-AS1 is a novel cancer-related lncRNA. Previous reports have illustrated that SATB2-AS1 is a critical regulator in some cancers. For example, some studies have confirmed that lncRNA SATB2-AS1 inhibits the growth of breast cancer cells by inhibiting miR-155-3p [9]. Xu M et al. also illustrated that lncRNA SATB2-AS1 abated colorectal cancer (CRC) metastasis and affected the microenvironment of tumor immune cells by regulating SATB2 [10]. Nevertheless, the specific mechanism of SATB2-AS1 in HCC is less understood.

MicroRNAs (miRNAs) are a type of endogenous noncoding small RNAs with 18–25 nucleotides in length. Previously, several studies have manifested that miRNAs exert a dominant role in the process of tumors [11]. For instance, miR-515-5p can suppress breast cancer by downregulating the mARK4/Hippo axis [12]. miR-296-5p impedes non-small cell lung cancer devolvement by targeting PLK1 [13]. MiR-3678-3p is a novel member of the miRNA family, located at 17q25.1, with a length of 94 bp. Recent studies have revealed that miR-3678-3p is involved in esophageal squamous cell carcinoma radiochemotherapy [14] and is associated with the epithelial-mesenchymal transition (EMT) status of colorectal cancer (CRC) [15]. Nevertheless, the specific role of miR-3678-3p in HCC has not been reported yet.

Genes associated with retinoid interferon-induced mortality (GRIM) is a kind of apoptosis protein. GRIM genes mediate IFN- and retinoic-acid (RA)-induced cell death [16]. GRIM-19, also known as NDUFA13, is one member of the GRIM family. Current studies state that GRIM-19 is implicated in the whole process of cell proliferation and apoptosis, and its upregulation weakens the aberrant proliferation and malignant transformation of tumor cells. For instance, GRIM-19 abates hypoxia-induced autophagy by inactivating STAT3/HIF-1α, thereby restraining CRC invasion and EMT [17]. Lin H et al. found that GRIM-19 suppressed prostate cancer cell proliferation and promoted apoptosis through the inhibition of miR-423-5p [18]. Therefore, we speculate that GRIM-19 is a tumor suppressor in HCC.

Here, we tried to investigate the potential role and mechanism of lncRNA SATB2-AS1 in HCC progression. SATB2-AS1 was downregulated in HCC cells and tissues and inhibited the proliferation and metastasis of HCC cells. A positive relationship between SATB2-AS1 and GRIM-19 was observed. miR-3678-3p was found as a downstream target of SATB2-AS1, and reduced GRIM-19 expression. Thus, we guessed that there is a novel regulatory axis, namely the SATB2-AS1-miR-3678-3p/GRIM-19/STAT3/HIF-1α axis, on HCC development. We hope this study provides a molecular reference for the clinical therapy and prognosis of HCC.

## Materials and methods

### Collection and treatment of clinical specimens

Eight-four HCC patients who underwent hepatectomy in Luzhou People’s Hospital from June 2012 to June 2018 were selected, and their tumor tissues were collected. None of the patients received preoperative chemotherapy and radiotherapy. The control samples were from the same patient's adjacent non-tumor tissues (more than 3 cm away from the surgical resection edge), and no tumor cells were discovered in the postoperative pathological examination. According to the world health organization (WHO) criteria, HCC was pathologically diagnosed. All specimens were removed and immediately preserved in − 196 ℃ liquid nitrogen until they were adopted for RNA extraction. The research ethics committee of Luzhou People’s Hospital approved our study, and all patients signed the informed consent.

### Cell culture and transfection

The American Type Culture Collection (ATCC, Rockville, MD, USA) provided human normal liver cell lines L-O2 and HCC cell lines (Huh7 and HCCLM3). These cells were cultured with the RPMI1640 medium (Thermo Fisher Scientific, MA, USA) containing 10% fetal bovine serum (FBS, Thermo Fisher Scientific, MA, USA) and 1% penicillin/streptomycin (Invitrogen, CA, USA) in an incubator (37 ℃, 5% CO_2_). During the logarithmic growth phase, the cells underwent trypsinization with 0.25% trypsin (Thermo Fisher, HyClone, Utah, USA).

SATB2-AS1 overexpression plasmids, GRIM-19 knockdown plasmids, miR-3678-3p mimics, and corresponding negative controls (Si-NC) were provided by GenePharma (Shanghai, China). Huh7 and HCCLM3 cells were inoculated in 24-well plates (3 × 10^5^ cells/well). After a 24-h incubation (37 ℃, 5% CO_2_), the cells were transfected using Lipofectamine^®^ 3000 (Invitrogen, Thermo Fisher Scientific, Inc.). The transfection efficiency was verified by reverse transcription-polymerase chain reaction (RT-PCR). Then, the cells were incubated at 37 °C for 24 h with 5% CO_2_.

### Reverse transcriptase polymerase chain reaction (RT-PCR)

The total cellular RNA was extracted with the TRIzol reagent (Invitrogen, Waltham, MA, USA). The extracted RNA was reversely transcribed into cDNA as per the manufacturer’s instructions with the PrimeScript™ RT Reagent kit (Invitrogen, Shanghai, China). The Bio-Rad CFX96 quantitative PCR system and SYBR were applied for RT-PCR following the manufacturer's specifications. RT-PCR was conducted with pre-denaturation at 95 °C for 5 min, denaturation at 95 °C for 15 s, and incubation at 60 °C for 30 s. GAPDH was the endogenous control for SATB2-AS1, and GRIM-19. U6 served as the internal control of miR-3678-3p and the other miRNAs. The relative expression of the detected genes was quantified by the 2^−∆∆Ct^ method. RT-PCR was conducted three times, and the primers were designed and synthesized by Guangzhou Ribo Biotechnology Co., Ltd. The primer sequences of the detected genes are shown in Table 1.

**Table 1:** Primer sequence of each gene

Gene name	Primer sequences (5` → 3`)
SATB2-AS1	Forward: CGAATCCCTTCCTCCTCTCC
	Reverse: TCGTCTTAGCCCTTTCCGTT
GRIM-19	Forward: CGCCGAGAATAACAAGACCG
	Reverse: GTGCTCAACGAACGCCATAT
miR-3678-3p	Forward: ACAGGCCCATTTGTCCCATA
	Reverse: CAGCCAACCTCAGAGAGACA
miR-1184	Forward: CCTGCAGCGACTTGATGGC
	Reverse: GAACATGTCTGCGTATCTC
miR-6105-3p	Forward: GCCGAGGGAGTTGCCAGGGCTGC
	Reverse: CAGTGCGTGTCGTGGAGT
miR-1205	Forward: CTGCAGGGTTTGCTTTGAGG
	Reverse: CTCCAGAACAGGGTTGACAGG
miR-17-3p	Forward: ATTACGGACTGCAGTGAAGGCAC
	Reverse: ATCCAGTGCAGGGTCCGAGG
miR-4713-3p	Forward: GCCGAGGTCCCCATTTTTCTCCC
	Reverse: CAGTGCGTGTCGTGGAGT
GAPDH	Forward: TGATCTTCATGGTCGACGGT
	Reverse: CCACGAGACCACCACCTACAACT
U6	Forward: CTCGCTTCGGCAGCACA
	Reverse: AACGCTTCACGAATTTGCGT

### Cell colony formation assay

Forty-eight hours after the transfection, the treated Huh7 and HCCLM3 cells were inoculated in 6-well plates (1000 cells/well) and cultured for 14 days. After fixing with 4% paraformaldehyde, the cell colonies were dyed with 0.5% crystal violet solution for 10 min and washed with PBS 3 times. Finally, the cell colonies were counted under a light microscope.

### EdU assay

EdU assay was implemented for detecting the proliferation of Huh7 and HCCLM3 cells according to the instructions of the BeyoClick™ EdU-488 Cell Proliferation Kit (Beyotime, Shanghai, China). The cells were inoculated on 24-well plates with 2 × 10^5^ cells per well. 200 μL of 5 μmol/L EdU working solution was added to each well and incubated for 2 h, and the cells were rinsed with PBS. The cells were fixed with 4% paraformaldehyde and then incubated with 200 μL of glycine (2 mg/mL) for 5 min and cleared with PBS on a shaker for 5 min. Afterward, 100 μL 0.5% Triton X-100 was added for permeability. Subsequently, the cells were incubated with the click additive solution (in the dark, RT, 30 min) and then stained with 1 × Hoechst 33342 DNA staining solution (in the dark, RT, 20 min). After washing with PBS, the cells were photographed and calculated under a fluorescence microscope (Olympus, Tokyo, Japan).

### Flow cytometry (FCM)

FCM (BD Biosciences, Franklin Lakes, NJ) was utilized to test Huh7 and HCCLM3 cells’ apoptosis. The double Annexin V/PI staining procedures were performed as required by the provider (Invitrogen). After double-staining, samples were analyzed via FCM.

### Transwell assay

Huh7 and HCCLM3 cells were treated with 0.25% trypsin, centrifuged, resuspended, and dispersed in each well of a 24-well culture plate. Transwell chambers (8 µm pore size; Corning, Beijing, China) were adopted for the migration and invasion experiment. 5 × 10^4^ transfected cells were placed in the upper chamber, and the Matrigel (BD, SanJose, USA) was precoated with the chambers in the invasion assay. 400 μL RPMI medium containing 10% FBS was added to the lower chamber. After incubation for 24 h at 37 °C, the non-migrated cells were removed. Transwell membranes were immobilized with 4% paraformaldehyde for 10 min and then dyed with 0.5% crystal violet. After washing with tap water, cell counting was made under an inverted microscope (Olympus, Tokyo, Japan). All tests were done three times.

### Western blot (WB)

After cell treatment, the medium was discarded. The protein lysis solution (Roche) was added, and the total protein was separated. Then, 50 μg of the total protein was taken and sampled on a 12% polyacrylamide gel and electrophoresed (100 V, 2 h). Afterward, the protein was transferred to polyvinylidene fluoride (PVDF) membranes. After being blocked with 5% skimmed milk at RT for 1 h, the membranes were rinsed with TBST 3 times (10 min each time) and incubated with the primary antibodies (1:1000) including anti-Bad (ab32445), anti-bcl2 (ab32124), anti-Bax (ab32503), anti-Caspase3 (ab32351), anti-p21 (ab109520), anti-Cyclin D (ab16663), anti-CDK4 (ab108357), anti-Cyclin E (ab33911), anti-CDK2 (ab32147), anti-GRIM-19 (ab110240), anti-E-cadherin (ab16505), anti-Vimentin (ab92547), anti-N-cadherin (ab18203), anti-p-STAT3 (ab76315), anti-STAT3 (ab68153), and anti-HIF-1α (ab179483) overnight at 4℃. After washing the membranes with TBST, we incubated them with horseradish peroxidase (HRP)-labeled goat-anti-rabbit IgG at RT (ab205718, 1:2500) for 1 h. The above antibodies were all from Abcam (Cambridge, UK). The membranes were rewashed 3 times with TBST (10 min each time). Finally, the ECL solution (Invitrogen) was utilized for protein band development and imaging, and Image J was employed to analyze the gray value of each protein.

### Immunohistochemistry (IHC)

After conventional paraffin embedding and sectioning (4 μm), HCC tissue samples were dewaxed with xylene, hydrated with gradient alcohol, and inactivated with 3% H_2_O_2_ for 10 min. 0.01 mol/L citrate sodium buffer was applied for microwave repair (pH = 6.0, 15 min). After the sections were blocked with 5% bovine serum albumin (BSA) for 20 min, they were incubated with the antibodies of anti-GRIM-19 (ab110240, Abcam, MA, USA), anti-Ki67 (ab15580, Abcam, MA, USA), anti-E-cadherin (ab16505, Abcam, MA, USA), and anti-Vimentin (ab92547, Abcam, MA, USA) at 4 ℃ overnight. The next day, the goat-anti-rabbit IgG was added and incubated at RT for 20 min. After washing with PBS, DAB was used for color development. After hematoxylin restaining, the sections were dehydrated, transparentized, mounted, and examined under a microscope. Image-Pro Plus (Media Cybem etics, America) was applied for analysis.

### Cellular immunofluorescence

The Huh7 and HCCLM3 cells were trypsinized and seeded in 24-well plates. After being incubated at 37 ℃ with 5% CO_2_ for 48 h, the cells were fixed with 4% paraformaldehyde for 20 min at RT, and their endogenous peroxidase was blocked with 3% H_2_O_2_ for 15 min. 0.5% Triton X-100 was added for permeability. The cells were then blocked with 5% goat serum for 1 h and incubated with the primary anti-GRIM-19 antibody (ab110240, 1:1000, Abcam, Cambridge, UK) overnight at 4 ℃. Subsequently, they were incubated with the Goat Anti-Mouse IgG H&L (Alexa Fluor^®^ 488) (ab150113, 1:200, Abcam, Cambridge, UK) for 1 h at RT. The nucleus was stained with the DAPI solution. The images were observed and collected under a fluorescence microscope.

### Fluorescence in situ hybridization (FISH) analysis

SATB2-AS1 and miR-3678-3p were captured by Cy3-labeled probes (RiboBio, Guangzhou, China) and Alexa 488-labeled probes (FOCOFISH, Guangzhou, China). The Fluorescent in situ Hybridization Kit (No. C10910, RiboBio, Guangzhou, China) was applied for FISH, according to the official guidelines. The nuclei were stained by DAPI. Subsequently, SATB2-AS1 and miR-3678-3p signals were detected with a confocal microscope (LSM 880 with Airyscan, Carl Zeiss, Germany).

### Dual-luciferase reporter assay

According to the binding site between SATB2-AS1 and miR-3678-3p, GRIM-19 and miR-3678-3p, the SATB2-AS1 or GRIM-19 3′UTR fragments containing miR-3678-3p target sites (wild-type and mutant-type) were inserted to pmirGLO dual-luciferase reporter vectors (Promega, Madison, WI), which were named as SATB2-AS1-WT/MUT and GRIM-19 3′UTR-WT/MUT. The vectors were co-transfected with miR-3678-3p mimics or NC mimics into Huh7 cells. Their luciferase intensity was verified with a dual-luciferase reporter assay system (Promega).

### RNA immunoprecipitation (RIP) assay

The Magna RIP™ RNA-Binding Protein Immunoprecipitation Kit (Millipore, Bedford, MA) was applied for RIP assay by using the anti-Ago2 antibody and anti-IgG antibody (negative control). The cultured cells were lysed, and then the lysates were treated with RIP buffer covering antibody-bound magnetic beads, followed by the analysis of the precipitated RNAs by RT-PCR.

### Tumor formation in nude mice

6-week-old BALB/c-nu nude female mice were provided by the animal center of Southwest Medical University. Huh7 stably transfected with SATB2-AS1 overexpressing plasmids or negative control group (2 × 10^6^ cells per nude mouse) were collected by 0.25% trypsin, then washed and resuspended in serum-free medium to make single-cell suspensions (2 × 10^7^ cells/mL). Then the cells were subcutaneously inoculated into mice for constructing a tumor formation model in vivo. Forty nude mice were divided into 4 groups, and 0.1 ml of cell suspension was injected subcutaneously into the left forelimb of each nude mouse. After the injection, the tumor volumes were calculated in the 1st week, 2nd week, 3rd week, 4th week, and 5th week. The weight of the tumor tissues derived from mice was measured.

### Statistical analysis

SPSS17.0 statistical software (SPSS Inc., Chicago, IL, USA) was adopted for analysis. Measurement data were presented as mean ± standard deviation (x ± sd), and the comparison between the two groups was made by *t-test or* chi-square test according to data type. The mean comparison between multiple groups was made by variance analysis. *P* < 0.05 indicated statistical significance.

## Results

### LncRNA SATB2-AS1 was downregulated in HCC tissues and cells

**Fig. 1:**
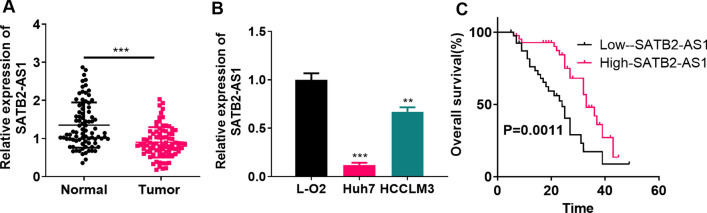
LncRNA SATB2-AS1 had lower expression in HCC tissues and cells. **A**: RT-PCR detected the SATB2-AS1 expression in HCC tissues and paracancerous non-tumor tissues. *** indicates *P* < 0.001 (vs. the Normal group). **B**: RT-PCR was implemented to verify the SATB2-AS1 profile in human normal hepatocytes and HCC cell lines (including Huh7 and HCCLM3). * indicates *P* < 0.05, ** indicates *P* < 0.01, *** indicates *P* < 0.001 (vs. L-O2 group). N = 3 **C**Prognostic analysis of SATB2-AS1 in HCC was performed using the K-M plotter assay

RT-PCR was performed to clarify the expression pattern of SATB2-AS1 in HCC tissues. As a result, SATB2-AS1 was downregulated in HCC tissues (vs. non-tumor tissues adjacent to carcinoma) (*P* < 0.05, Fig. 1A). Similarly, the SATB2-AS1 expression in HCC cell lines (Huh7 and HCCLM3) was considerably impeded compared with that in normal human liver cells L-O2 (*P* < 0.05, Fig. 1B). Moreover, patients with lower SATB2-AS1 levels had higher TNM stages (P = 0.008) and a higher risk of recurrence (P = 0.032) (Table 2). Besides, the survival time of patients with lower SATB2-AS1 expression was shorter than those with higher SATB2-AS1 expression (P = 0.0011, Fig. 1C). The above results suggested that SATB2-AS1 plays a carcinogenic role in HCC.

**Table 2:** Relationship between the SATB2-AS1 expression and HCC patients’ clinical features

Clinical parameters		Patients (n)	Expression of SATB2-AS1		P-value
			High-SATB2-AS1	Low-SATB2-AS1	
Total		84	42	42	
Age (years)	< 70	34	15	19	0.374
	≥ 70	50	27	23	
Gender	Male	45	25	20	0.274
	Female	39	17	22	
Tumor size (cm)	< 5	24	14	10	0.334
	≥ 5	60	28	32	
Serum AFP(ng/ml)	< 20	33	15	18	0.503
	≥ 20	51	27	24	
Cirrhosis of the liver	Yes	58	28	30	0.637
	No	26	14	12	
TNM stage	I-II	34	23	11	0.008*
	III-IV	50	19	31	
Tumor recurrence	Yes	59	25	34	0.032*
	No	25	17	8	

*P < 0.05 was statistically significant

### Overexpressing SATB2-AS1 suppressed the malignant phenotypes of HCC cells

**Fig. 2:**
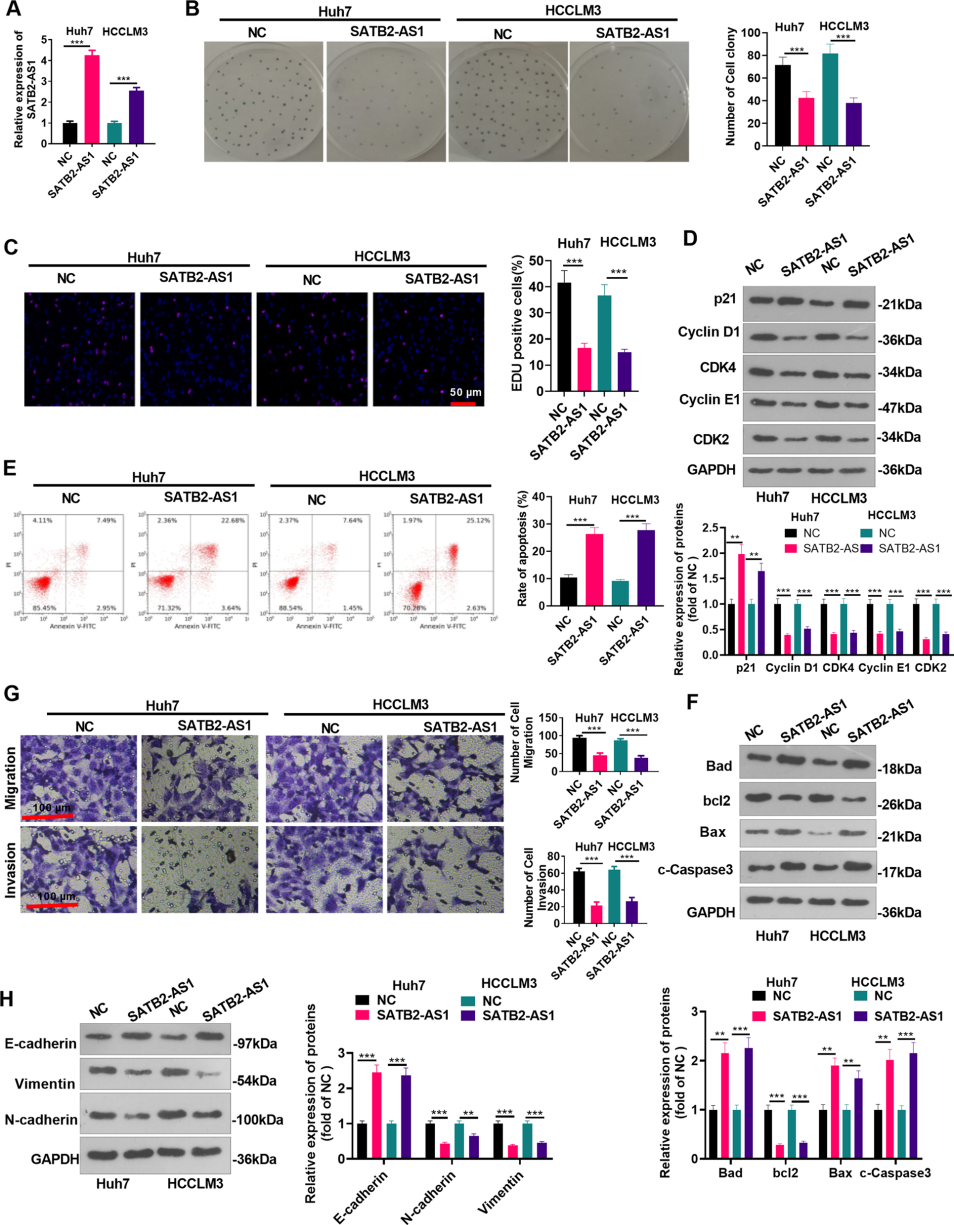
Overexpressing SATB2-AS1 abated the malignant phenotypes of HCC. **A** SATB2-AS1 overexpression model was constructed in Huh7 and HCCLM3 to probe the influence of SATB2-AS1 on HCC progression. A: RT-PCR detected the SATB2-AS1 level. **B**, **C**: HCC cell proliferation was determined by the colony formation assay (**B**) and EdU experiment (**C**). Scale bar = 50 μm. **D** Western blot was conducted for detecting cell cycle-related proteins (including p21, Cyclin D1, CDK4, Cyclin E1, and CDK2) levels in HCC cells. **E**. Flow cytometry (FCM) was adopted to monitor the HCC cell apoptosis rate. **F** Western blot was conducted for detecting apoptosis-related proteins (including Bad, bcl2, Bax, and cleaved Caspase3) levels in HCC cells. **G**. Transwell assay was utilized to assess HCC cell migration and invasion. Scale bar = 100 μm. **H **:WB was applied to test the expression of E-cadherin, Vimentin, and N-cadherin. ** indicates *P* < 0.01, *** indicates *P* < 0.001. N = 3

We constructed the SATB2-AS1 overexpression model in HCC cell lines (Huh7 and HCCLM3) to probe the influence of SATB2-AS1 on HCC (*P* < 0.05, Fig. 2A). The colony formation experiment and EdU staining were applied to verify cell proliferation. It was found that the number of colony-forming cells and EDU-positive cells were significantly reduced after overexpressing SATB2-AS1 (*P* < 0.05, Fig. 2B, C). Western blot was performed for detecting cell cycle-related proteins, including p21, Cyclin D1, CDK4, Cyclin E1, and CDK2. The data showed that SATB2-AS1 upregulation promoted p21, whereas inhibited Cyclin D1, CDK4, Cyclin E1, and CDK2 in the two HCC cells (Fig. 2D). FCM and western blot were used for evaluating cell apoptosis. The results manifested that the intervention of SATB2-AS1 elevated the apoptosis rate (*P* < 0.05 vs. NC group, Fig. 2E), reduced the bcl2 level and promoted Bax, Bad, and cleaved Caspase3 expression (Fig. 2F). Transwell assay confirmed that after forced upregulation of SATB2-AS1, cell migration and invasion were reduced (*P* < 0.05 vs.NC group, Fig. 2G). Furthermore, WB was implemented to compare the expression of EMT-related proteins, and it was found that overexpressing SATB2-AS1 up-regulated E-cadherin but down-regulated Vimentin and N-cadherin (*P* < 0.05, Fig. 2H). The above results indicated that SATB2-AS1 was involved in HCC progression, and overexpressing SATB2-AS1 abated HCC growth, migration, invasion, and induced apoptosis.

### LncRNA SATB2-AS1 repressed the growth and metastasis of HCC in vivo

**Fig. 3:**
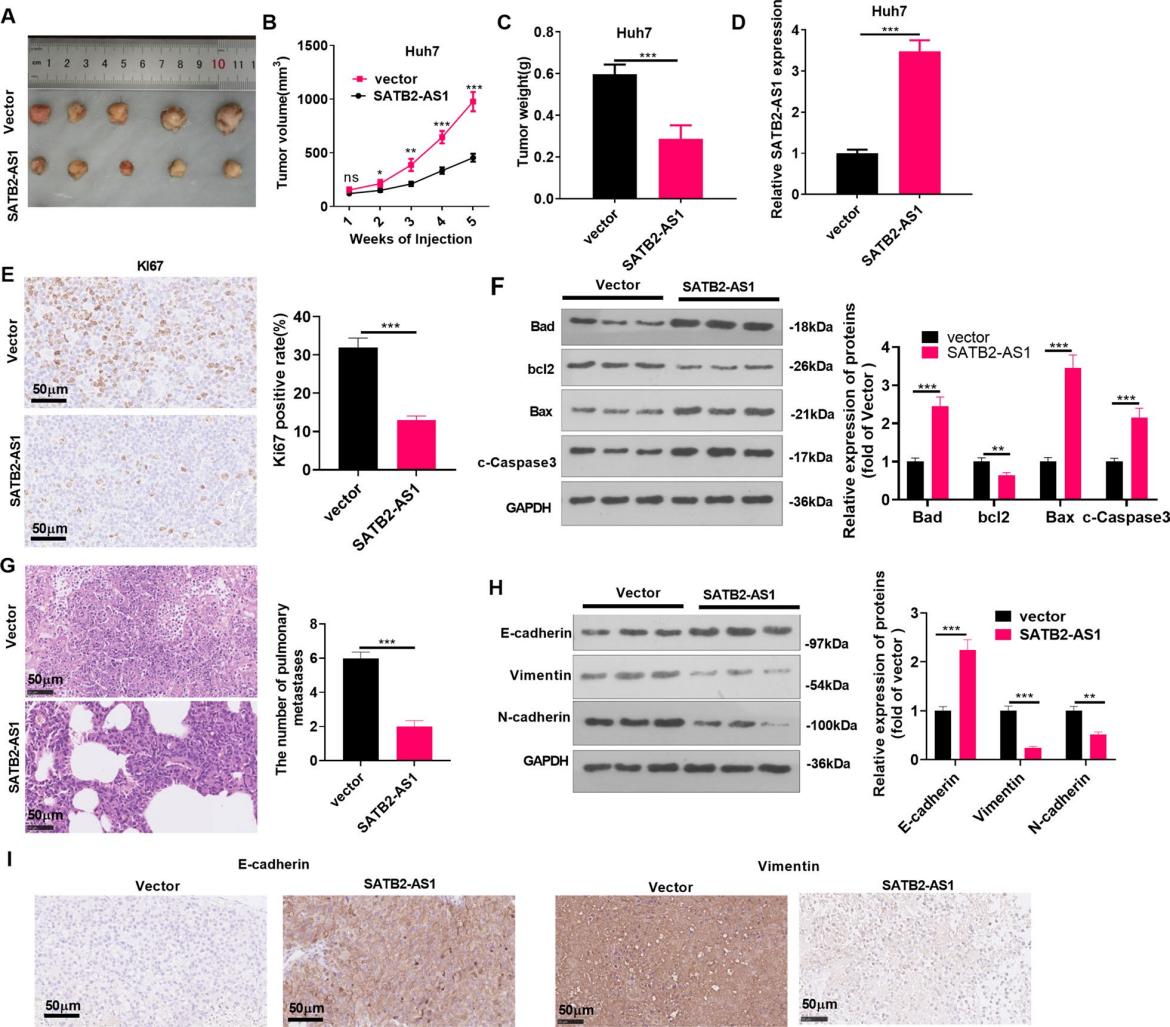
LncRNA SATB2-AS1 repressed HCC growth and metastasis in vivo. Huh7 cells transfected with negative vector or SATB2-AS1 overexpression plasmids were subjected to the subcutaneous xenografted tumor experiment on nude mice. **A**, **C**: The nude mice were sacrificed after 5 weeks, and the subcutaneous tumor nodules were removed. The volume (**B**) and weight (**C**) of tumor nodules were calculated. **D**. The SATB2-AS1 expression in vivo was monitored by RT-PCR. **E**: IHC was implemented to examine the number of Ki67-positive cells in Huh7. Scale bar = 50 μm. **F**: Western blot was conducted for detecting apoptosis-related proteins (including Bad, bcl2, Bax, and cleaved Caspase3) levels in the tumor tissues. **G**. HE staining was applied to test lung metastasis of HCC, and the number of lung metastasis was counted. **H**: The profiles of E-cadherin, Vimentin, and N-cadherin were compared by WB. **I**: IHC was applied to calculate positive cells for E-cadherin and Vimentin. Scale bar = 50 μm. ns indicates *P* > 0.05, * indicates *P* < 0.05, ** indicates *P* < 0.01, *** indicates *P* < 0.001. N = 5

We constructed SATB2-AS1 overexpression models in Huh7 cell lines, and an *in-vivo* experiment was conducted to test cell growth. It was found that the tumor volume and weight and SATB2-AS1 level in the SATB2-AS1 overexpressing group were heightened (vs. the vector group) (*P* < 0.05, Fig. 3A-D). IHC results illustrated that the number of Ki67-positive cells declined significantly after SATB2-AS1 overexpression, indicating a significant decrease in tumor proliferation (*P* < 0.05, Fig. 3E). Western blot results indicated that Bcl2 was downregulated in the SATB2-AS1 group, whereas Bad, Bax and cleaved Caspase3 were upregulated (compared with the vector group, p < 0.05, Fig. 3F). Meanwhile, lung metastases from HCC decreased significantly (*P* < 0.05, Fig. 3G). The expression of EMT-related proteins was compared by WB, and the results revealed that overexpressing SATB2-AS1 elevated the E-cadherin expression but impeded Vimentin and N-cadherin (*P* < 0.05, Fig. 3H). IHC unveiled that compared with the vector group, the SATB2-AS1 group had more E-cadherin-positive cells and fewer Vimentin-positive cells (Fig. 3I). These findings further confirmed that overexpressing SATB2-AS1 promoted HCC growth.

### LncRNA SATB2-AS1 facilitated GRIM-19 expression and inactivated the STAT3/HIF-1α pathway

**Fig. 4:**
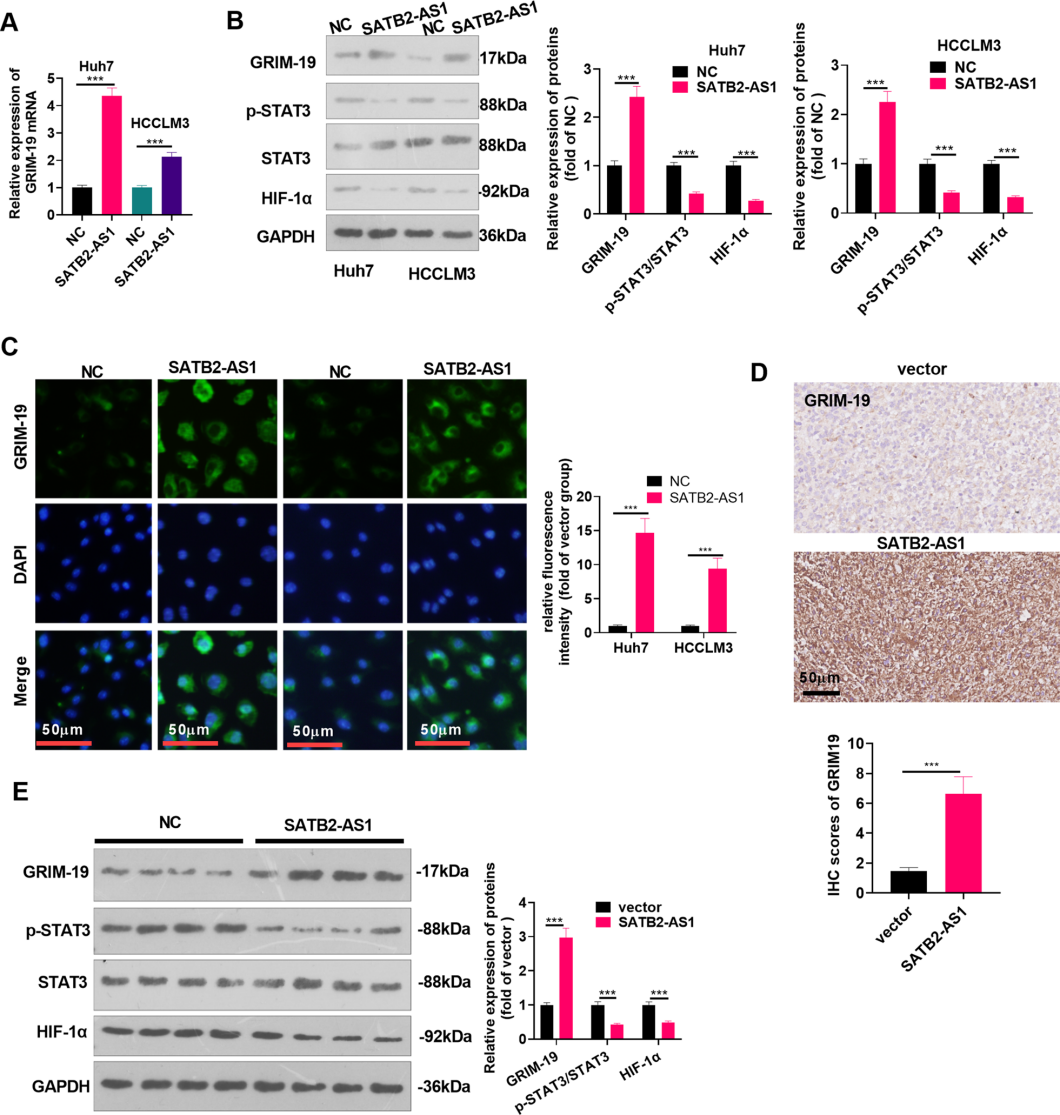
LncRNA SATB2-AS1 facilitated GRIM-19 and inactivated the STAT3/HIF-1α pathway. The SATB2-AS1 overexpression model was established in Huh7 and HCCLM3 cells. **A**, **B**: The expression of GRIM-19 mRNA and the GRIM-19/STAT3/HIF-1α pathway were measured by RT-PCR (**A**) and WB (**B**), respectively. **C**: Cellular immunofluorescence was conducted to examine the fluorescence intensity of GRIM-19 (marked by green fluorescence). Scale bar = 50 μm. **D**: IHC was applied to determine the expression of GRIM-19 in the tumor tissues. Scale bar = 50 μm. IHC score was counted. **E**: WB was implemented to examine the GRIM-19/STAT3/HIF-1α pathway expression in the tumor tissues. *** indicates *P* < 0.001. N = 3

We detected GRIM-19 mRNA and STAT3/HIF-1α expression in Huh7 and HCCLM3 cells by RT-PCR and WB, respectively. As a result, the GRIM-19 was significantly up-regulated after SATB2-AS1 overexpression (*P* < 0.05, Fig. 4A), while STAT3/HIF-1α was down-regulated (*P* < 0.05, Fig. 4B). The GRIM-19 profile was further determined by cellular immunofluorescence, and the results manifested that the fluorescence intensity of GRIM-19 was more significant after overexpressing SATB2-AS1 (*P* < 0.05 compared with the NC group, Fig. 4C). In vivo, IHC verified that the staining score of GRIM-19 was significantly enhanced after overexpressing SATB2-AS1 (*P* < 0.05, Fig. 4D). WB results testified that GRIM-19 was significantly upregulated, while the STAT3/HIF-1α pathway was downregulated in the SATB2-AS1 group (vs. the vector group) (*P* < 0.05, Fig. 4E). These conclusions illustrated that SATB2-AS1 modulated the GRIM-19/STAT3/HIF-1α pathway expression.

### Inhibiting GRIM-19 activated the STAT3/HIF-1α pathway and accelerated HCC devolvement

**Fig. 5:**
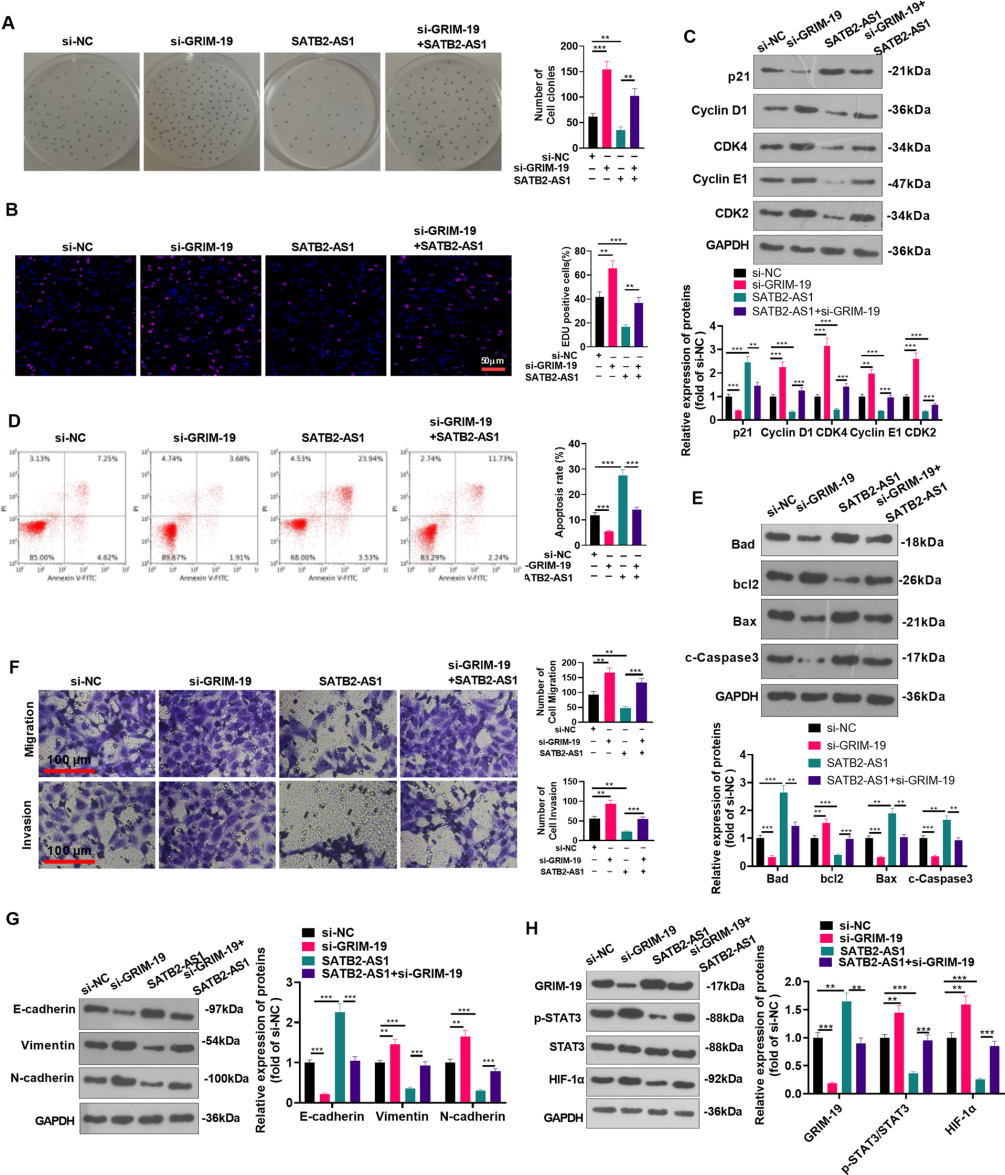
Inhibiting GRIM-19 activated the STAT3/HIF-1α pathway and strengthened HCC progression. GRIM-19 knockdown plasmids and/or SATB2-AS1 overexpression plasmids were transfected into Huh7 cells. **A**, **B**: Colony formation experiment and EdU staining were utilized to detect cell proliferation. Scale bar = 50 μm. **C**: Western blot was conducted for detecting cell cycle-related proteins (including p21, Cyclin D1, CDK4, Cyclin E1, and CDK2) levels in HCC cells. **D**. Flow cytometry (FCM) was adopted to monitor the HCC cell apoptosis rate. **E**: Western blot was conducted for detecting apoptosis-related proteins (including Bad, bcl2, Bax, cleaved Caspase3) levels in HCC cells. **F**: Huh7 cell migration and invasion were tested by Transwell assay. Scale bar = 100 μm. **G**: WB was implemented to determine the levels of EMT-related proteins (E-cadherin, Vimentin, and N-cadherin). **H**: The GRIM-19/STAT3/HIF-1α expression in Huh7 cells was measured by WB. * indicates *P* < 0.05, ** indicates *P* < 0.01, *** indicates *P* < 0.001. N = 3

We transfected the GRIM-19 knockdown plasmids and/or SATB2-AS1 overexpression plasmids into Huh7 to explore the influence of inhibiting GRIM-19 on HCC evolvement and its specific mechanism. First, cell proliferation was tested by the colony formation experiment and EdU staining. As a result, it increased significantly after GRIM-19 knockdown, while overexpressing SATB2-AS1 exerted the opposite effect. In contrast, compared with the SATB2-AS1 group, the cell viability of the SATB2-AS1 + si-GRIM-19 group was significantly heightened (*P* < 0.05, Fig. 5A, B). GRIM-19 knockdown reduced p21 level, and promoted Cyclin D1, CDK4, Cyclin E1, CDK2. SATB2-AS1 upregulation exerted the opposite effects. Compared with SATB2-AS1 group, SATB2-AS1 + si-GRIM-19 group had reduced p21, but increased Cyclin D1, CDK4, Cyclin E1, CDK2 levels (p < 0.05, Fig. 5C). Additionally, FCM analysis and Western blot demonstrated that the percentage of apoptosis declined after GRIM-19 knockdown, while SATB2-AS1 overexpression led to completely opposite results (vs. the si-NC group). Meanwhile, the combined utilization of SATB2-AS1 and si-GRIM-19 reversed the function of SATB2-AS1 (*P* < 0.05, Fig. 5D, E). Transwell assay illustrated that cell migration and invasion were enhanced after knocking down GRIM-19, while they were dampened by overexpressing SATB2-AS1 (vs. the si-NC group). Compared with the SATB2-AS1 group, cell migration and invasion of the SATB2-AS1 + si-GRIM-19 group were strengthened (*P* < 0.05, Fig. 5F). Furthermore, WB found that the E-cadherin level was repressed, and the expression of Vimentin and N-cadherin was strengthened after GRIM-19 knockdown while overexpressing SATB2-AS1 exerted the opposite function. Besides, compared with the SATB2-AS1 group, E-cadherin was lowly expressed, but Vimentin and N-cadherin were overexpressed in the SATB2-AS1 + si-GRIM-19 group (*P* < 0.05, Fig. 5G). Moreover, WB results manifested that the si-GRIM-19 group had downregulated GRIM-19 and up-regulated STAT3/HIF-1α, while the SATB2-AS1 group had the opposite result (vs. the si-NC group). On the contrary, compared with the SATB2-AS1 group, the GRIM-19 level in Huh7 cells in the SATB2-AS1 + si-GRIM-19 group was downregulated, and the STAT3/HIF-1α pathway profile was upregulated (*P* < 0.05, Fig. 5H). These findings unveiled that inhibiting GRIM-19 activated the STAT3/HIF-1α pathway and enhanced the malignant phenotypes of HCC.

### MiR-3678-3p was the target of SATB2-AS1 and targeted GRIM-19

**Fig. 6:**
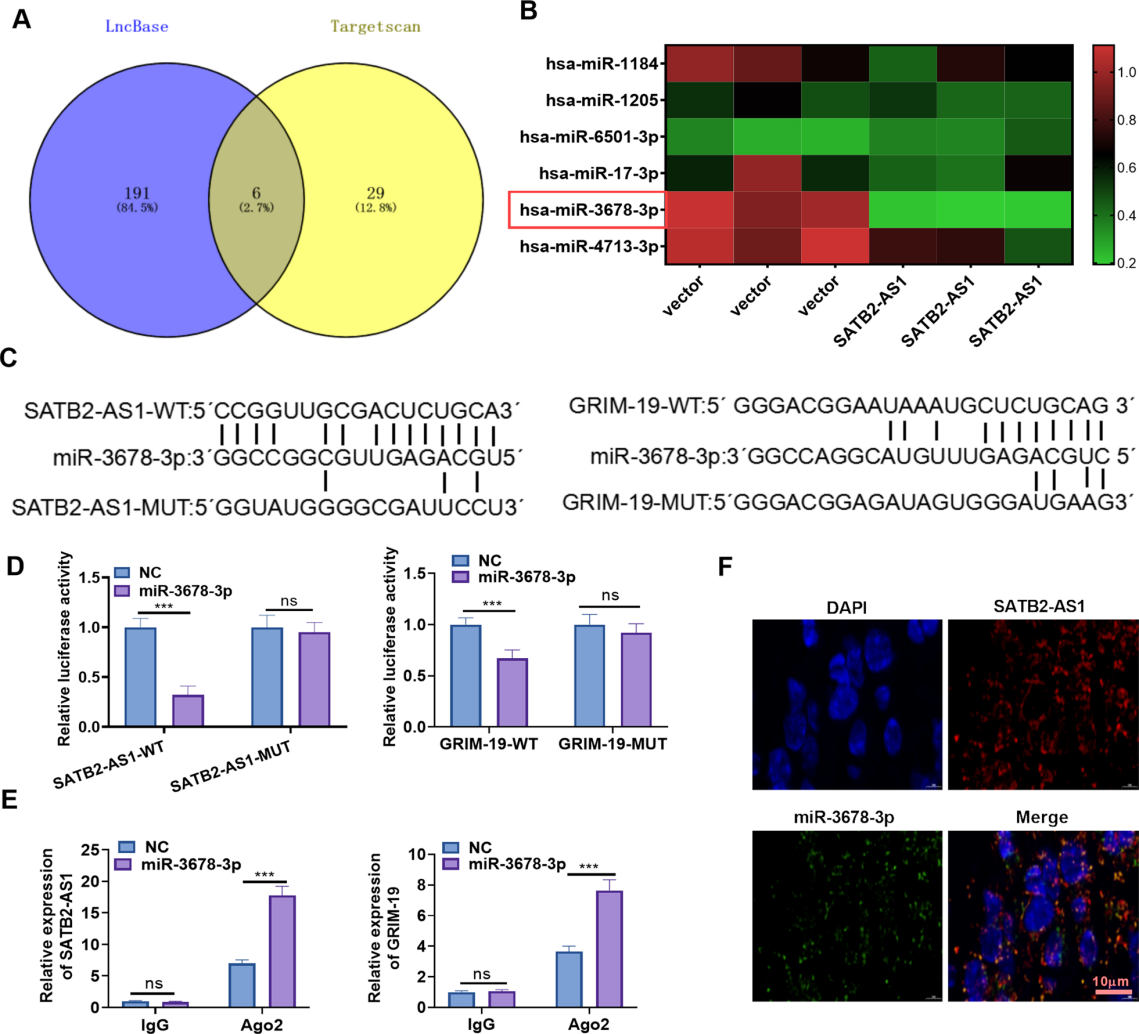
MiR-3678-3p was targeted by SATB2-AS1. The miRNA targets of SATB2-AS1 and GRIM-19 were predicted by LncBase Predicted v.2 (http://carolina.imis.athena-innovation.gr/diana_tools/web/index.php?r=lncbasev2%2Findex) and Targetscan (https://www.kegg.jp/), respectively. **A**: Venn diagram analysis was utilized to verify the common miRNA targets between SATB2-AS1 and GRIM-19. Six miRNAs (including has-miR-1184, has-miR-1205, has-miR-6501-3p, has-miR-17-3p, has-miR-3678-3p, has-miR-4713-3p) were common targets to SATB2-AS1 and GRIM-19. **B**. RT-PCR was conducted for detecting including has-miR-1184, has-miR-1205, has-miR-6501-3p, has-miR-17-3p, has-miR-3678-3p, has-miR-4713-3p in Huh7 cells transfected with vector or SATB2-AS1. **C**: The binding sites between miR-3678-3p and SATB2-AS1, miR-3678-3p, and GRIM-19. **D**, **E**: SATB2-AS1-WT/MUT and GRIM-19 3′UTR-WT/MUT vectors, along with miR-3678-3p mimics or NC mimics were transfected into Huh7 cells. Dual-luciferase reporter assay was performed. **E**. Huh7 cells were transfected with miR-3678-3p mimics, and an RIP assay was conducted. The enrichment levels of SATB2-AS1, GRIM-19, and miR-3678-3p in the lysates were detected by RT-PCR. **F**: FISH unveiled colocalization between miR-3678-3p (green) and SATB2-AS1 (red) in Huh7 cells. SATB2-AS1 probes and miR-3678-3p probes were labeled with Alexa Fluor 555 and Alexa Fluor 488, respectively. DAPI was employed for nuclei staining; Scale bar = 10 μm. ns indicates *P* > 0.05, *** indicates *P* < 0.001. N = 3

Inspired by the lncRNA-miRNA-mRNA regulatory axis, we probed the miRNA target of SATB2-AS1 and GRIM-19 through LncBase Predicted v.2 and Targetscan. It turned out that 6 miRNAs (including has-miR-1184, has-miR-1205, has-miR-6501-3p, has-miR-17-3p, has-miR-3678-3p, has-miR-4713-3p) were common targets of SATB2-AS1 and GRIM-19 (Fig. 6A). Next, we applied RT-PCR to compare the profile of these 6 miRNAs in SATB2-AS1 overexpressed cells. As a result, miR-3678-3p was most significantly down-regulated (Fig. 6B). In LncBase Predicted v. 2, we found that SATB2-AS1 targeted the 3'UTR end of miR-3678-3p. Additionally, we discovered that GRIM-19 had a targeted binding relationship with miR-3678-3p in the Targetscan database (Fig. 6C). The dual-luciferase reporter assay results illustrated that miR-3678-3p abated the luciferase activity of SATB2-AS1-WT and GRIM-19-WT, but it had no significant impact on that of cells transfected with SATB2-AS1-MUT and GRIM-19-MUT (*P* > 0.05, Fig. 6D). RIP assay results manifested that after the miR-3678-3p mimics transfection, the amount of SATB2-AS1 and GRIM-19 precipitated in the Ago2 antibody group was more than that in the IgG group, confirming that SATB2-AS1 and GRIM-19 bound to Ago2 through miR-3678-3p (*P* < 0.05, Fig. 6E). Furthermore, the RNA FISH assay indicated a high degree of colocalization between SATB2-AS1 and miR-3678-3p in HCC cells (*P* < 0.05, Fig. 6F). These findings illustrated that there was a targeted binding association between SATB2-AS1 and miR-3678-3p, GRIM-19 and miR-3678-3p.

### MiR-3678-3p promoted the malignant phenotypes of HCC

**Fig. 7:**
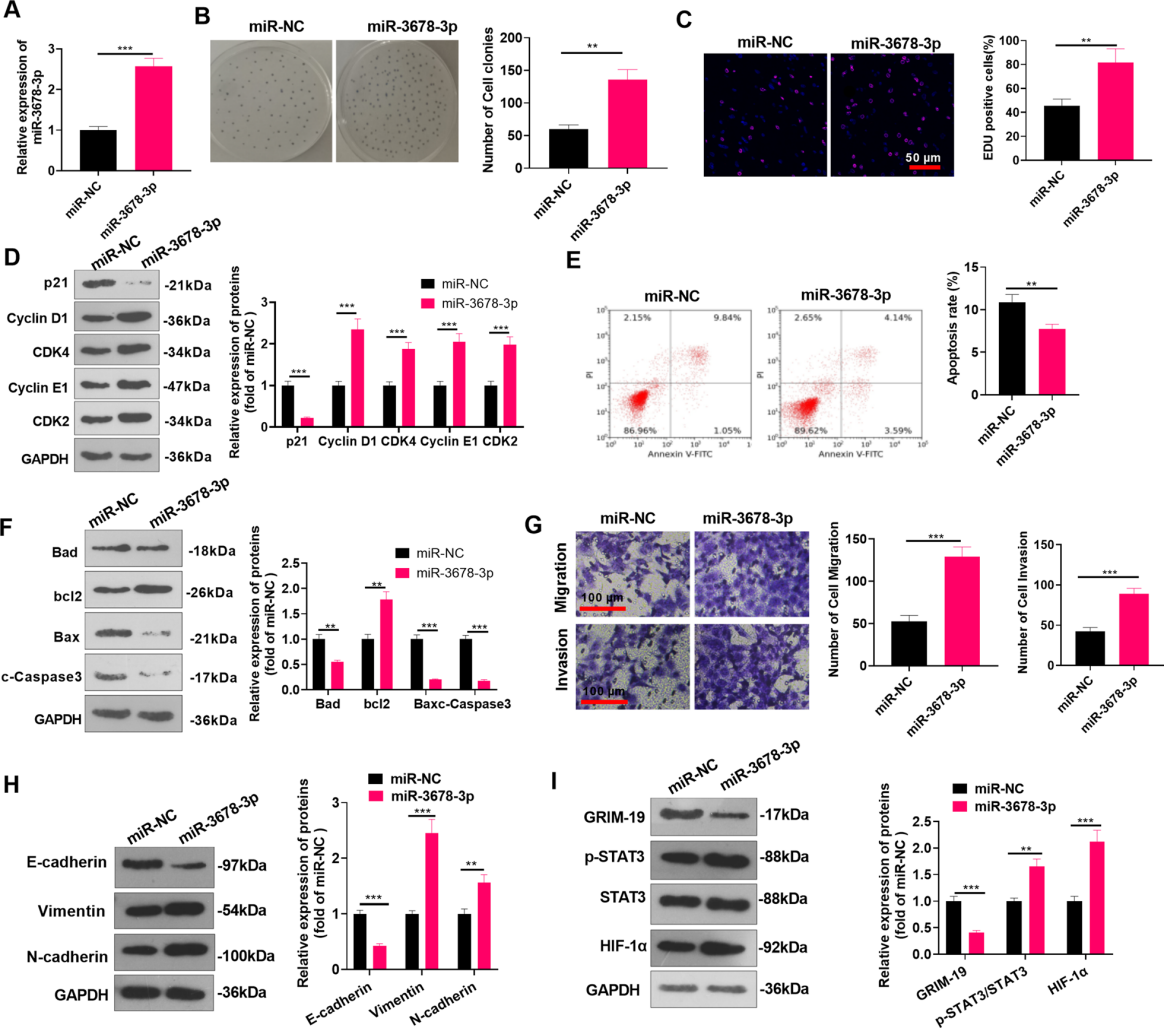
MiR-3678-3p accelerated the malignant phenotypes of HCC. The miR-3678-3p mimic was transfected in Huh7 cells. **A**: RT-PCR detected the transfection effect of miR-3678-3p. **B**, **C**: Colony formation experiment and EdU staining were applied to examine cell proliferation. Scale bar = 50 μm. **D**: Western blot was conducted for detecting cell cycle-related proteins (including p21, Cyclin D1, CDK4, Cyclin E1, and CDK2) levels in HCC cells. **E**. Flow cytometry (FCM) was adopted to monitor the HCC cell apoptosis rate. **F**: Western blot was conducted for detecting apoptosis-related proteins (including Bad, bcl2, Bax, cleaved Caspase3) levels in HCC cells. **G**: Huh7 cell migration and invasion were monitored by Transwell assay. Scale bar = 100 μm. **H**: WB was implemented to test the profiles of EMT-related proteins. **I**: WB was conducted to measure the GRIM-19/STAT3/HIF-1α pathway level in Huh7 cells. ** indicates *P* < 0.01, *** indicates *P* < 0.001. N = 3

We transfected miR-3678-3p mimics into Huh7 cells to explore the impact of miR-3678-3p on HCC. First, RT-PCR was implemented to inspect the transfection efficiency (*P* < 0.05, Fig. 7A). The colony formation experiment and EdU assay revealed that the number of colony-forming cells and EdU-positive cells in the miR-3678-3p group was elevated (vs. the miR-NC group) (*P* < 0.05, Fig. 7B, C). miR-3678-3p upregulation reduced p21 level and enhanced Cyclin D1, CDK4, Cyclin E1, and CDK2 expressions (*P* < 0.05 vs.miR-NC group, Fig. 7D). The results of FCM and Western blot manifested that the apoptosis rate, protein levels of Bax, Bad, and cleaved Caspase3 expressions were mitigated, whereas bcl2 was promoted after the miR-3678-3p mimic transfection (*P* < 0.05 vs.miR-NC group, Fig. 7E, F). Transwell assay revealed that the miR-3678-3p group had elevated cell migration and invasion (vs. the miR-NC group) (*P* < 0.05 vs.miR-NC group, Fig. 7 G). Furthermore, WB confirmed that E-cadherin was down-regulated, while Vimentin and N-cadherin were upregulated after the miR-3678-3p mimic transfection (*P* < 0.05 vs.miR-NC group, Fig. 7 H). WB also demonstrated that GRIM-19 was downregulated, and STAT3/HIF-1α was overexpressed after the miR-3678-3p mimics transfection (*P* < 0.05 vs.miR-NC group, Fig. 7I). These results testified that miR-3678-3p strengthened HCC cell proliferation and metastasis and inhibited cell apoptosis potentially by mediating the GRIM-19/STAT3/HIF-1α pathway.

### SATB2-AS1 repressed the carcinogenic effects mediated by miR-3678-3p

**Fig. 8:**
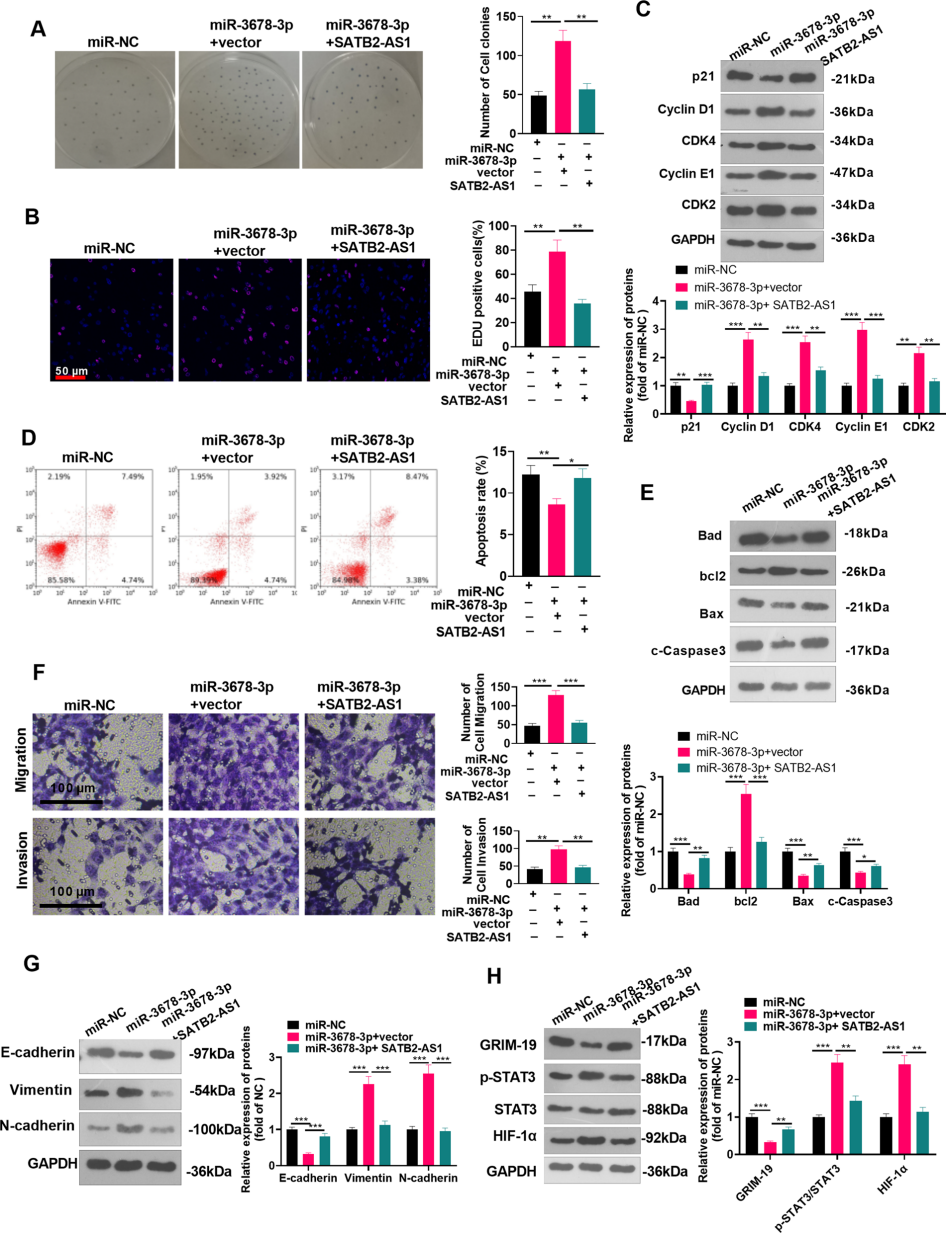
SATB2-AS1 impeded the carcinogenic effect mediated by miR-3678-3p. SATB2-AS1 was overexpressed in Huh7 cells transfected with miR-3678-3p mimics. **A**, **B**: Colony formation experiment and EdU staining were applied to test cell proliferation. Scale bar = 50 μm. **C**: Western blot was conducted for detecting cell cycle-related proteins (including p21, Cyclin D1, CDK4, Cyclin E1, and CDK2) levels in HCC cells. **D**. Flow cytometry (FCM) was adopted to monitor the HCC cell apoptosis rate. **E**: Western blot was conducted for detecting apoptosis-related proteins (including Bad, bcl2, Bax, and cleaved Caspase3) levels in HCC cells. **F**: Huh7 cell migration and invasion were monitored by Transwell assay. Scale bar = 100 μm. **G**. The levels of EMT-related proteins were compared by WB. **H**: WB was performed to test the expression of the GRIM-19/STAT3/HIF-1α pathway in Huh7 cells. * indicates *P* < 0.05, ** indicates *P* < 0.01, *** indicates *P* < 0.001. N = 3

SATB2-AS1-overexpressed Huh7 cells were transfected with miR-3678-3p mimics to explore the influence of SATB2-AS1 on the miR-3678-3p-mediated carcinogenic effect. First, the colony formation experiment and EdU staining were employed to verify cell proliferation. It was found that the number of colony-forming cells and EdU-positive cells in the miR-3678-3p + vector group was elevated (vs. the miR-NC group). However, after transfection of the SATB2-AS1 overexpression plasmids, the cell viability decreased significantly (*P* < 0.05 vs. miR-3678-3p + vector group, Fig. 8A, B). Western blot data showed that SATB2-AS1 overexpression enhanced p21, and reduced Cyclin D1, CDK4, Cyclin E1, and CDK2 expressions (*P* < 0.05 vs.miR-3678-3p + vector group, Fig. 8C). The results of FCM and western blot showed that the transfection of SATB2-AS1 overexpression plasmids resulted in enhanced apoptosis rate and protein levels of Bad, Bax, and cleaved Caspase3, and reduced bcl2 level compared with the miR-3678-3p group) (*P* < 0.05, Fig. 8D, E). Transwell assay manifested that compared with the miR-NC group, cell migration and invasion in the miR-3678-3p + vector group were significantly increased. However, they were reduced after the transfection of the SATB2-AS1 overexpression plasmid (vs. the miR-3678-3p + vector group) (*P* < 0.05, Fig. 8F). Furthermore, WB testified that E-cadherin was downregulated, while Vimentin and N-cadherin were significantly upregulated in the miR-3678-3p + vector group compared with that of the NC group. In contrast, the results after transfection of SATB2-AS1 overexpression plasmids were completely opposite (vs. the miR-3678-3p + vector group) (*P* < 0.05, Fig. 8G). Also, WB results manifested that GRIM-19 was downregulated, and the STAT3/HIF-1α pathway expression was upregulated after the miR-3678-3p mimic was transfected in Huh7 cells. On the contrary, the results were completely opposite after transfection of SATB2-AS1 overexpression plasmids (vs. the miR-3678-3p + vector group) (*P* < 0.05, Fig. 8H). The above results testified that miR-3678-3p facilitated the malignant phenotypes of HCC cells, while SATB2-AS1 significantly weakened the effect.

### STAT3 repressed SATB2-AS1 and promoted miR-3678-3p

**Fig. 9:**
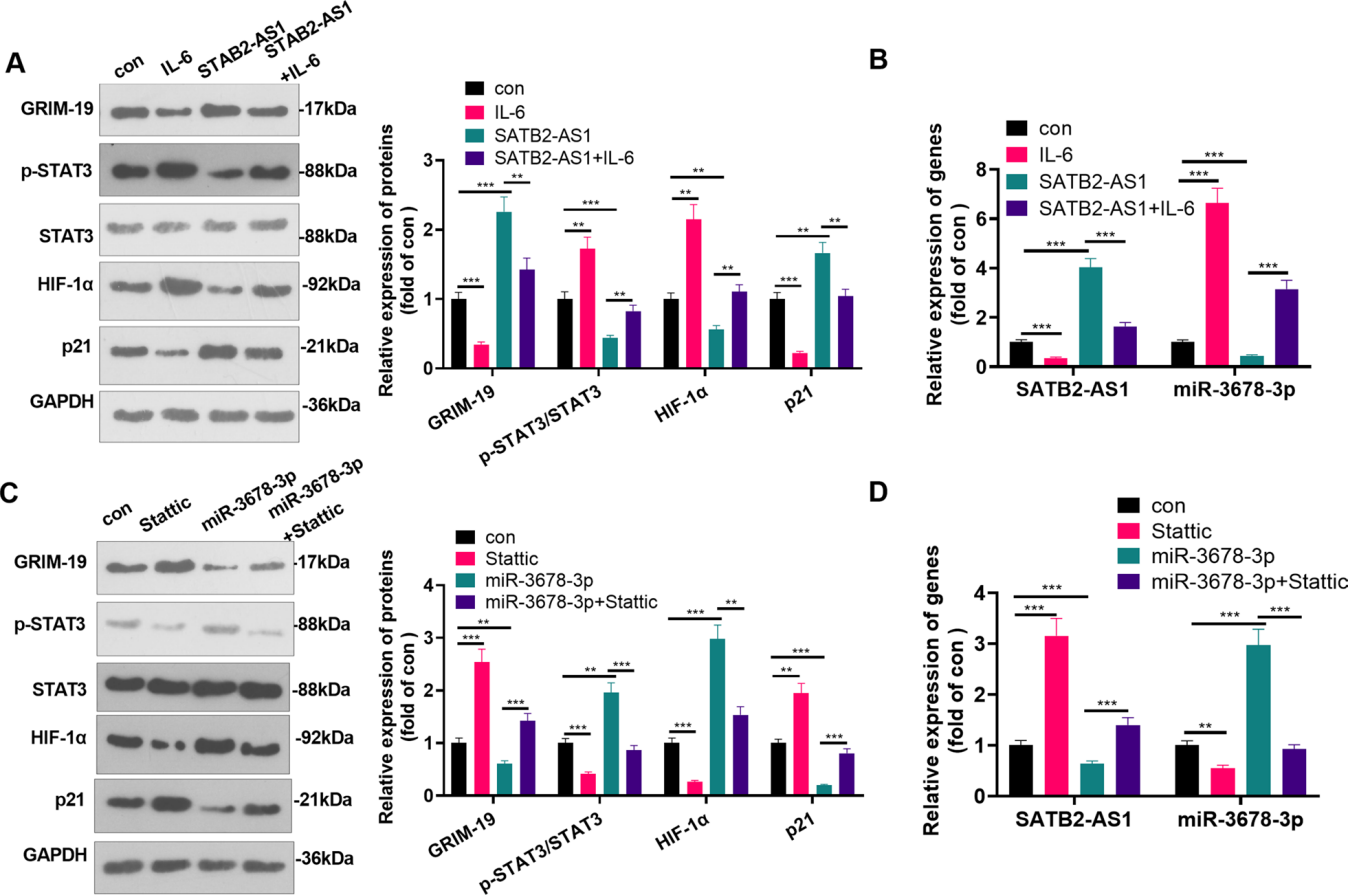
STAT3 repressed SATB2-AS1 and promoted miR-3678-3p. Huh7 cells were treated with IL-6 (20 ng/ml) or Stattic (5 μM) for 24 h. **A**. WB was performed to test the expression of the GRIM-19/STAT3/HIF-1α pathway in Huh7 cells. **B**. RT-PCR was used for detecting SATB2-AS1 and miR-3678-3p levels. **C**. WB was performed to test the expression of the GRIM-19/STAT3/HIF-1α pathway in Huh7 cells. **D**. RT-PCR was used for detecting SATB2-AS1 and miR-3678-3p levels. ** indicates *P* < 0.01, *** indicates *P* < 0.001. N = 3

**Fig. 10:**
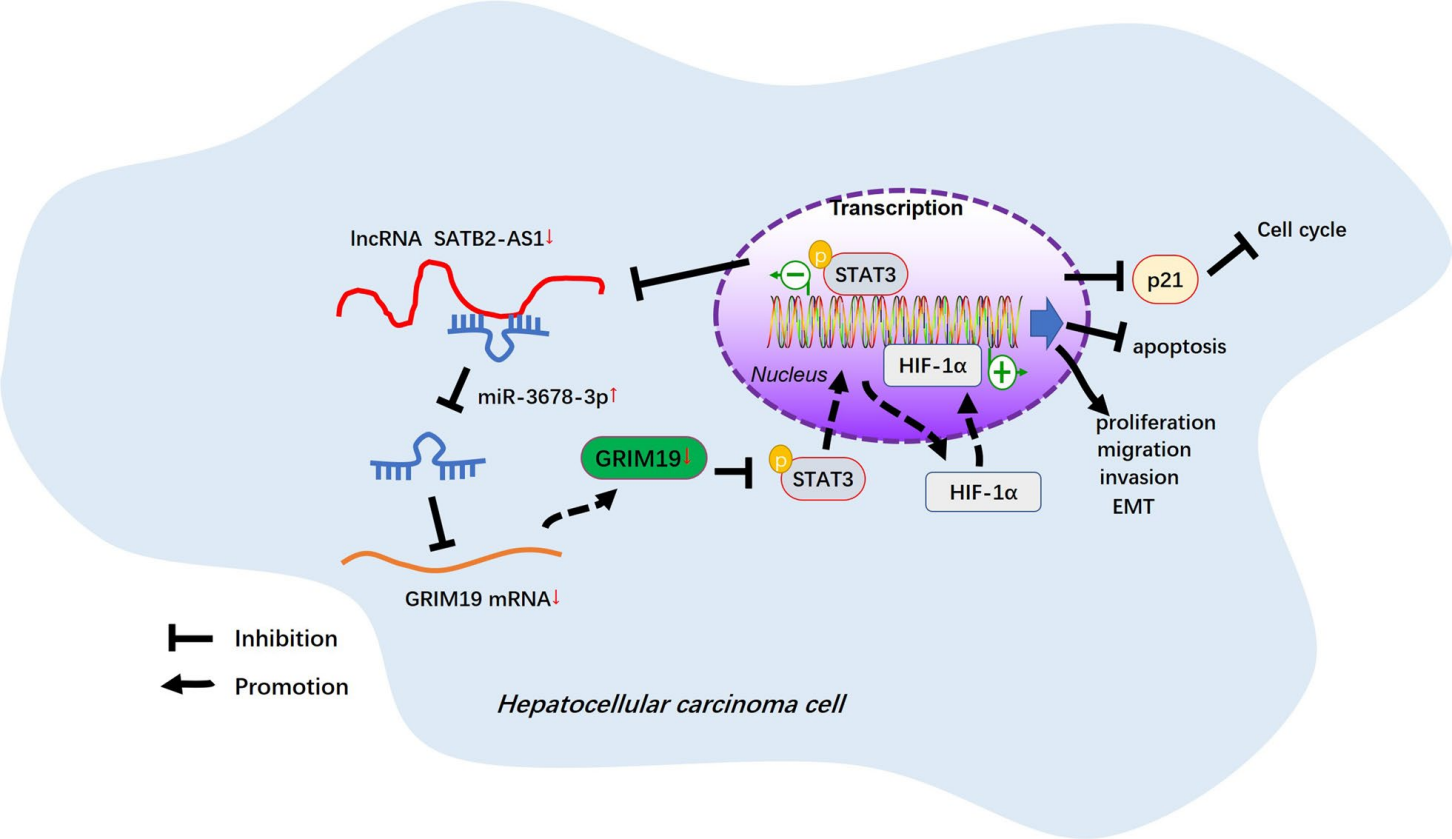
The schematic diagram. SATB2-AS1 promoted GRIM-19 by targeting miR-3678-3p. GRIM-19 inactivates STAT3/HIF-1α pathway and induces apoptosis, cell cycle arrest, proliferation, and metastasis inhibition

For investigating the potential role of STAT3 in mediating SATB2-AS1 and miR-3678-3p, we treated Huh7 cells with IL-6 (20 ng/ml) for activating the STAT3 pathway, and a STAT3 inhibitor Stattic (5 μM) was used for inhibiting STAT3 pathway. Western blot and RT-PCR results showed that compared with the con group, IL-6 treatment reduced GRIM-19, p21, and SATB2-AS1, while promoted p-STAT3, HIF-1α, and miR-3678-3p levels (Fig. 9A, B). Followed by SATB2-AS1 overexpression, GRIM-19, p21, and SATB2-AS1 were enhanced, and p-STAT3, HIF-1α, and miR-3678-3p levels were significantly downregulated (compared with IL-6 group, Fig. 9A, B). On the other hand, Stattic administration enhanced GRIM-19, p21, and SATB2-AS1 levels, but inhibited p-STAT3, HIF-1α, and miR-3678-3p levels (Fig. 9C, D). However, forced miR-3678-3p upregulation reduced GRIM-19, p21, and SATB2-AS1 level, and promoted p-STAT3, HIF-1α and miR-3678-3p levels (Fig. 9C, D). Therefore, we believed that STAT3 had a feedback role on SATB2-AS1/miR-3678-3p/GRIM-19 axis (Fig. 10).

## Discussion

HCC is among the most lethal tumors globally, and its morbidity and mortality are still on the rise [1, 5]. Moreover, HCC is a chemotherapy-resistant tumor with a worse survival rate [19]. Therefore, it’s crucial to find targeted therapeutic targets. LncRNAs are regulatory RNAs discovered in recent years, which have attracted growing attention in tumor biology research [20]. Previous studies have shown that lncRNA SATB2-AS1 is involved in various tumors, including CRC [21, 22] and osteosarcoma [23]. Here, we found that SATB2-AS1 was significantly lowly expressed in HCC tissues and cells. Functional experiments proved that SATB2-AS1 dampened HCC cell proliferation and metastasis and expedited its apoptosis, thus inactivating the miR-3678-3p/GRIM-19/STAT3/HIF-1α pathway. This research has further improved the molecular mechanism of HCC and confirmed that SATB2-AS1 inhibited the progression of HCC.

Recently, emerging studies have unveiled that lncRNAs manipulate ceRNAs of other transcripts by competing with miRNAs. Current reports have found that diversified lncRNAs regulate the expression of related proteins through competitive binding with miRNAs to regulate HCC progression. For example, LncRNA SCRG1 serves as the ceRNA of miR-26a to limit its inhibitory effect on SKP2, thereby inducing HCC cell proliferation and migration in vivo and in vitro [24]. Liu Z et al. found that lncRNA PRR34-AS1 is overexpressed in HCC tissues and cell lines. It upregulates FOXO3 by targeting miR-498, thereby promoting HCC cell proliferation, migration, and invasion, and abating cell apoptosis [25]. Besides, Liu X et al. stated that lncRNA TMPO-AS1 is upregulated in HCC tissues and cell lines. It facilitates cell proliferation, and stemness and represses cell apoptosis by targeting the miR-429/GOT1 axis, which aggravates HCC [26]. Based on the above research, we were curious about whether SATB2-AS1 has a targeting relationship with a specific miRNA and then plays a corresponding regulatory role in HCC. Through bioinformatics analysis, we discovered that there is a conserved binding site of miR-3678-3p on SATB2-AS1. The dual-luciferase experiment and RIP method confirmed that there was a targeting correlation between SATB2-AS1 and miR-3678-3p. Meanwhile, overexpressing SATB2-AS1 in Huh7 cells hampered miR-3678-3p-mediated carcinogenic effects. In effect, overexpressing SATB2-AS1 inhibited HCC progression, while the transfection of miR-3678-3p mimics heightened the malignant phenotypes of HCC. These findings indicate that SATB2-AS1 is a miRNA sponge to bind and regulates miR-3678-3p.

GRIM-19 is a tumor suppressor gene. It has been reported that GRIM-19 is downregulated in HCC patients with liver capsule and microvascular infiltration, and its downregulation induces EMT [27]. Moreover, GRIM-19 plays an anti-tumor role in HCC by negatively regulating PI3K/AKT pathway [28]. Multiple studies have manifested that down-regulation of GRIM-19 motivates HIF-1α synthesis in a STAT3-dependent way [29]. For instance, Kong D et al. stated that GRIM-19 overexpression represses the proliferation and invasion of orthotopically implanted HCC by reversing the STAT3 pathway activation [30]. Besides, knocking down GRIM-19 causes excessive activation of p-STAT3 and facilitates HCC development [31]. More importantly, Zhang J et al. found that GRIM-19 abates hypoxia-induced CRC invasion and EMT by abating the HIF-1α/STAT3 pathway [17]. Moreover, GRIM-19 inhibits the aerobic glycolysis, cell proliferation, and tumorigenesis of head and neck squamous cell carcinoma by inhibiting HIF-1α/STAT3 [32]. Consistent with the above conclusions, this study found that SATB2-AS1 promotes GRIM-19 and inactivates STAT3/HIF-1α in Huh7 cells. Meanwhile, GRIM-19 knockdown activates the STAT3/HIF-1α pathway and aggravates HCC development. These results indicated that GRIM-19, as a tumor suppressor gene, hampers HCC progression by inactivating the STAT3/HIF-1α pathway.

STAT3/HIF-1α pathway has been regarded as a vital signaling pathway in controlling cell proliferation, apoptosis and cell cycle [33, 34]. HIF-1α expression was promoted followed by enhanced STAT3 phosphorylation. p53, p21, Bcl-2, which serve as crucial genes in mediating apoptosis and cell cycle, are dependently regulated by HIF-1α under hypoxia/hypoglycaemia [35, 36]. We determined apoptosis related proteins (including Bad, bcl2, Bax and cleaved Caspase3) and cell cycle-related proteins (including p21, Cyclin D1, CDK4, Cyclin E1, and CDK2) levels in HCC cells, and found that SATB2-AS1 promoted Bad, Bax, cleaved Caspase3, and p21 levels in HCC cells, but reduced bcl2, Cyclin D1, CDK4, Cyclin E1, CDK2 expressions. We believed that SATB2-AS1 overexpression promoted apoptosis and induced cell cycle arrest due to the inactivation of the STAT3/HIF-1a pathway. However, a larger cohort of HCC samples is needed to verify the clinical significance of lncRNA SATB2-AS1 gene expression in HCC diagnosis.

## Conclusion

Overall, our study confirmed that SATB2-AS1 was knocked down in HCC, and its overexpression impeded the proliferation and metastasis of HCC. This study clarified that SATB2-AS1, as one of the good prognostic factors of HCC, affects HCC progression by regulating the miR-3678-3p/GRIM-19/STAT3/HIF-1α pathway expression, providing a novel intervention target for the clinical treatment and prognosis of HCC (Fig. 10). Nevertheless, further molecular mechanisms should be probed.

## Data Availability

The data sets used and analyzed during the current study are available from the corresponding author on reasonable request.
